# Hypertensive retinopathy as a masquerade neuroretinitis in a child: a case report

**DOI:** 10.1186/s12886-026-04791-z

**Published:** 2026-05-05

**Authors:** Sarah Gabison, Rym Khellaf, Carl Arndt, Aurélie Bérot, Guillaume Silva, Nathalie Bednarek, Christine Pietrement, Thomas Ferreira de Moura

**Affiliations:** 1 Service d’ophtalmologie, Université de Reims Champagne-Ardenne, CHU de Reims, Reims, 51100 France; 2 Pédiatrie générale et spécialisée, Université de Reims Champagne-Ardenne, CHU Reims, Reims, 51100 France; 3 CRESTIC, Université de Reims Champagne Ardenne, Reims, France; 4 Service de Médecine Néonatale et Réanimation Pédiatrique, Université de Reims Champagne-Ardenne, CHU de Reims, Reims, France; 5 Extracellular Matrix and Cell Dynamics Unit (MEDyC) UMR, CNRS, University of Reims Champagne-Ardenne, Reims, France; 6 UMR-S 1320, Université Reims Champagne-Ardenne, CardioVir, Reims, 51100 France; 7 Service d’ophtalmologie, Hôpital Robert Debré, rue du Général Koenig, Reims, 51100 France

**Keywords:** Pediatric hypertension, Hypertensive retinopathy, Vesicoureteral reflux, High blood pressure

## Abstract

**Background:**

High blood pressure in children is rare and often underdiagnosed, but it can cause irreversible damage to target organs. Ocular signs of hypertensive retinopathy are nonspecific and may resemble those of neuroretinitis or papilledema, which can delay diagnosis.

**Case presentation:**

A 9-year-old girl presenting with bilateral visual loss was referred to our emergency department. Best-corrected visual acuity was 20/200 in the right eye and counting fingers in the left. Fundus examination revealed bilateral optic disc swelling with a macular star and flame-shaped hemorrhages. Infectious work-up and neuroimaging were inconclusive. Reassessment showed severe hypertension (165/118 mmHg), hypokalemia, impaired renal and cardiac function. Plasma renin and aldosterone were markedly elevated. Imaging revealed a hypoplastic left kidney with subtotal renal artery stenosis and high-grade vesicoureteral reflux, contributing only 7% of total renal function on DMSA scintigraphy. Antihypertensive therapy with amlodipine, enalapril, and acebutolol normalized blood pressure, improved renal and cardiac function, and restored full visual acuity within six months. A left nephrectomy was subsequently performed to improve long-term blood pressure control.

**Conclusion:**

This case demonstrates the importance of systematic blood pressure measurement in children with disc edema or neuroretinitis-like features. Severe hypertension should be considered early on. Pediatric guidelines recommend blood pressure targets below the 90th percentile with ACE inhibitors, angiotensin receptor blockers, calcium channel blockers, or thiazide diuretics as first-line agents. Nephrectomy may be indicated in cases of refractory hypertension when a hypoplastic kidney contributes less than 10% of overall function.

## Background

Pediatric hypertension is an uncommon but potentially serious condition that often remains undiagnosed until end-organ damage becomes evident. By definition, systolic blood pressure exceeding the 95th percentile for age, sex, and height is considered diagnostic of pediatric hypertension [1]. When unrecognized or untreated, it can lead to long-term cardiovascular, renal, and ocular complications. Fundoscopic findings may resemble a wide range of retinal pathologies, including cotton wool spots, flame-shaped hemorrhages, and in more severe cases, pseudo-papilledema, pseudo-neuroretinitis, or hypertensive chorioretinopathy [2]. Because clinical signs may be subtle or misleading, systematic blood pressure assessment is essential in any pediatric patient presenting with bilateral optic disc swelling. We report the case of a 9-year-old girl in whom severe renovascular hypertension mimicked bilateral neuroretinitis, resulting in delayed diagnosis and multisystemic involvement.

## Case presentation

**Fig. 1:**
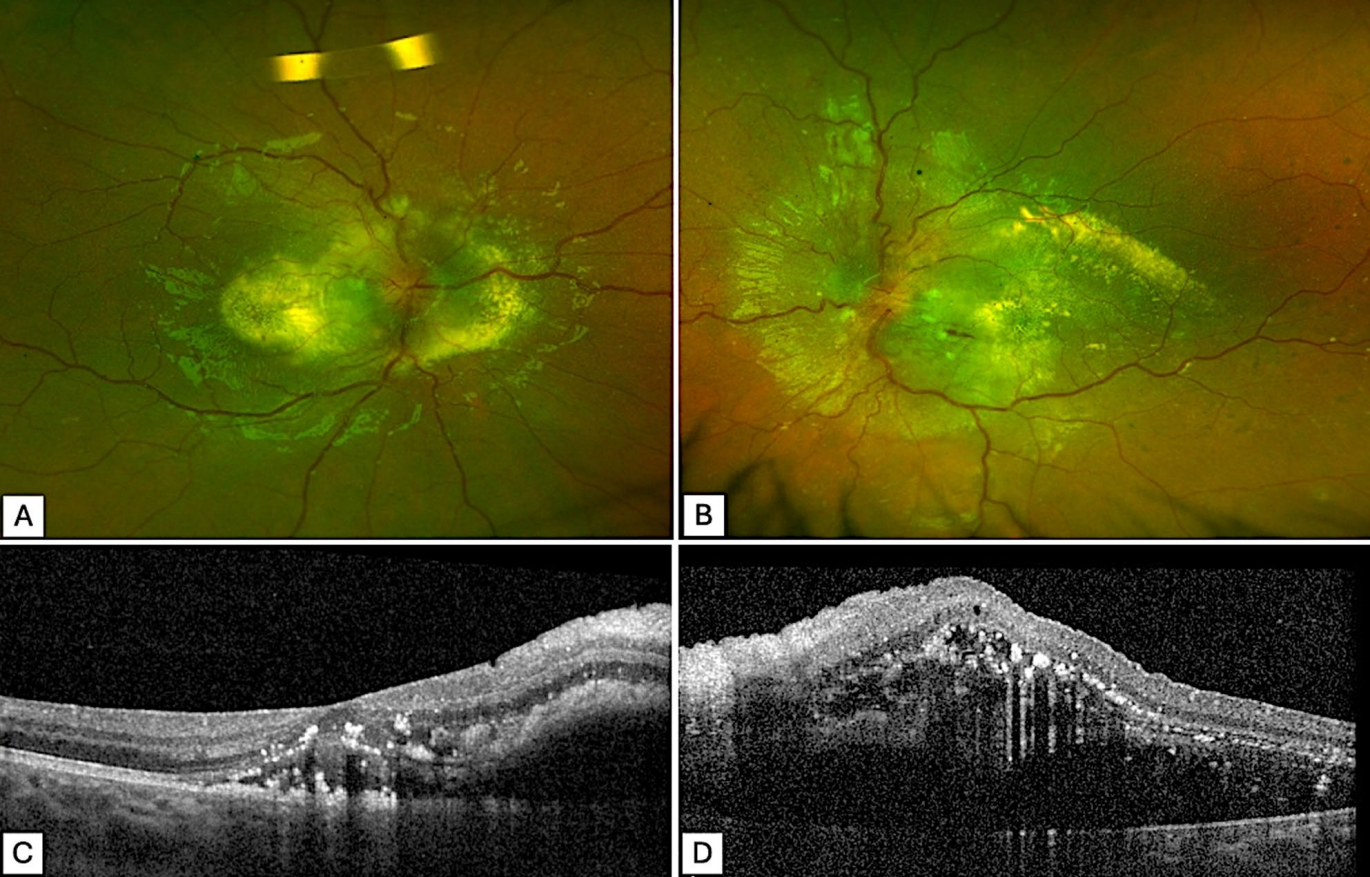
Initial fundus and OCT with clinical signs of neuroretinitis like features: **A-B**: Ultra-wide-field retinophotographs of the right (**A**) and left (**B**) eyes showing vascular tortuosity, bilateral macular star-shaped oedema associated with optic disc swelling. **C-D**: Macula optical coherence tomography (OCT) images of the right (**C**) and left eye (**D**) show the presence of serous detachment and intraretinal hyperreflective exudates

A 9-year-old girl was referred to the ophthalmology emergency department for a history of bilateral blurred vision associated with headaches and morning nausea. Her past medical history was unremarkable. Best-corrected visual acuity (BCVA) was 20/200 in the right eye and limited to counting fingers in the left eye. Fundus examination revealed bilateral disc edema with extensive macular star-shaped exudates, and flame-shaped hemorrhages. Macular optical coherence tomography (OCT) showed extensive intraretinal hyperreflective exudates and bilateral serous retinal detachment (Fig. 1). Indocyanine green (ICG) and fluorescein angiography confirmed optic disc swelling without signs of inflammation or choroidal ischemia.

Given the initial suspicion of neuroretinitis or intracranial hypertension, the patient was admitted to the pediatric department for a global check-up. Clinical examination revealed a systolic murmur without signs of heart failure. Blood pressure was initially recorded at 107/72 mmHg during the first hospitalization. Brain MRI showed FLAIR hypersignals in the putaminal area without features of idiopathic intracranial hypertension. Initial blood tests proved negative, particularly serology tests for bartonellosis, rickettsiosis, and toxocariasis.

**Fig. 2:**
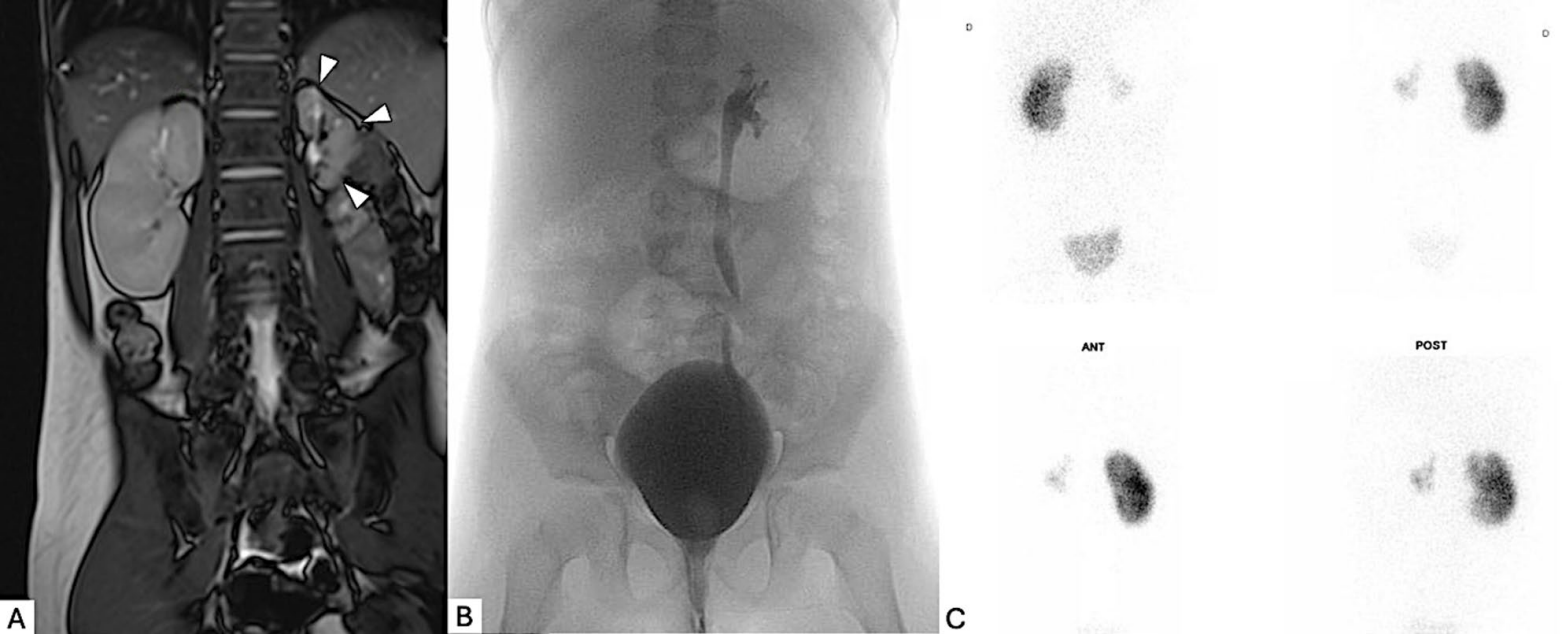
Multimodal imaging of unilateral renal hypoplasia with associated arterial stenosis. **A**: Coronal T2 MRI showing marked reduction in the size of the left kidney consistent with hypoplasia (white arrow), unlike the right kidney, which is of normal size. **B**: Cystography showing dilatation of the left ureter with reflux of contrast medium into the hypoplastic kidney, consistent with high-grade left vesicoureteral reflux. **C**: DMSA scintigraphy (anterior and posterior views) revealing decreased perfusion and tracer uptake in the left kidney compared to the right kidney

On the control blood tests, were found a persistent hypokalemia (2.8 mmol/L), an acute kidney injury with elevated serum creatinine (92 µmol/L, estimated glomerular filtration rate (eGFR) of 47 mL/min/1.73 m²) and a hypoalbuminemia (27 g/L). Urinalysis demonstrated severe proteinuria (5,587 mg/L, with a protein-to-creatinine ratio of 689 mg/mmol) without hematuria. Considering these findings, her blood pressure was re-measured and found to be markedly elevated at 165/118 mmHg. Given the suspicion of secondary hypertension, an endocrine and metabolic workup was initiated. Plasma aldosterone was elevated at 5 540 pmol/L (normal 144–676,9 pmol/L), and plasma renin activity was significantly increased at 1 083.3 ng/L in the supine position (normal < 30 ng/L). Cortisol levels were within normal limits., Plasma and urinary metanephrines were measured and tumors such as pheochromocytoma or other catecholamine-secreting tumors were ruled out. Renal ultrasound and magnetic resonance angiography revealed a hypoplastic left kidney with subtotal occlusion of the mid-segment of the left renal artery. The stenosis was associated with severe left vesicoureteral reflux. A DMSA renal scintigraphy showed that the left kidney contributed only 7% to overall renal function (Fig. 2).

Transthoracic echocardiography demonstrated concentric left ventricular hypertrophy with a reduced ejection fraction of 49%, consistent with hypertensive cardiomyopathy. These results led to the diagnosis of renovascular hypertension with systemic repercussions. Antihypertensive therapy was initiated with a combination of amlodipine, acebutolol, and enalapril.

Over the following weeks, the patient’s blood pressure normalized (around 116/66 mmHg), and cardiac function improved significantly. At one-month follow-up, ejection fraction had increased to over 60%, GFR improved to 62 mL/min, serum potassium had normalized, and proteinuria had markedly decreased to 48 mg/mmol.

**Fig. 3:**
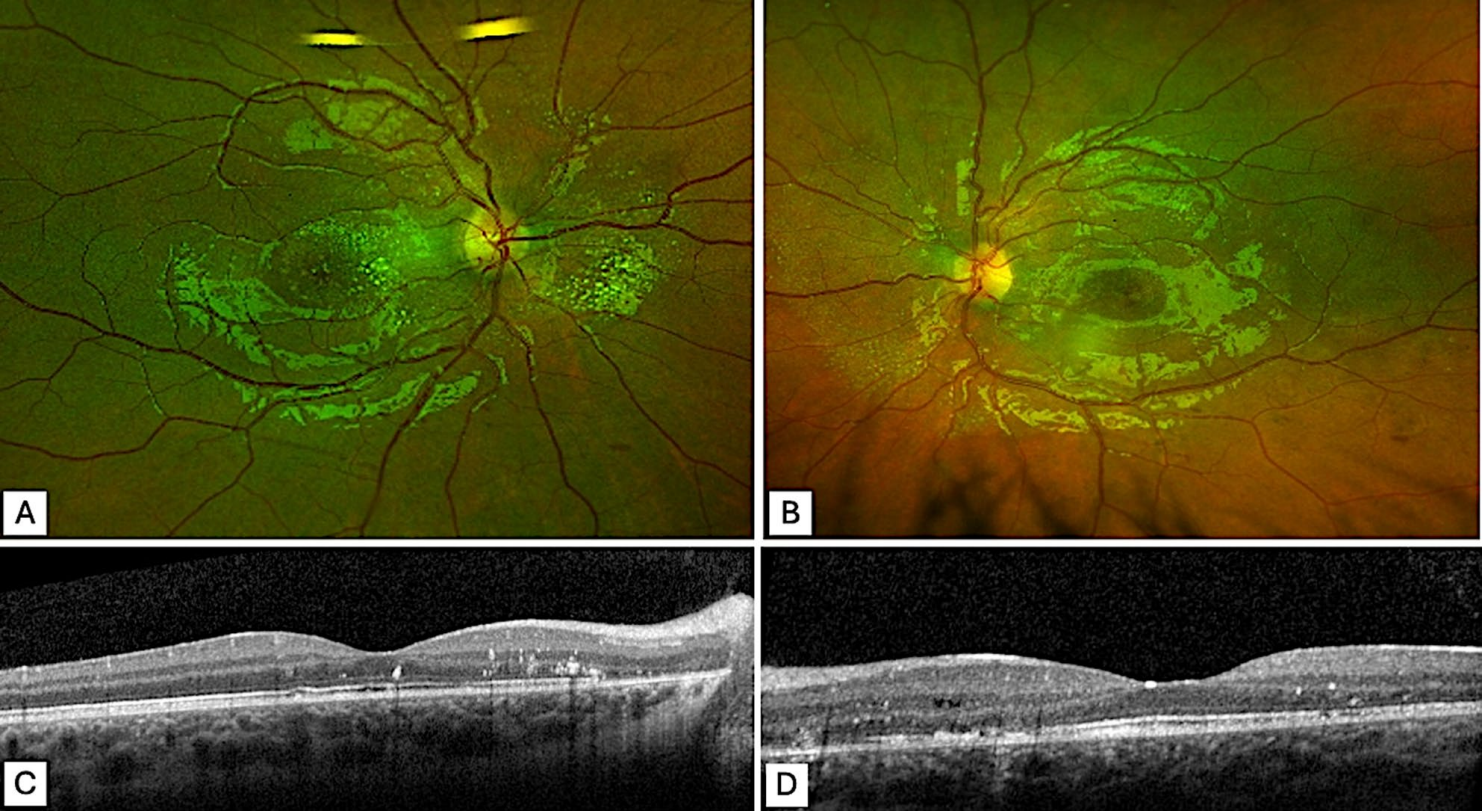
Fundus photographs and macular OCT 6 months after the start of antihypertensive treatment. **A-B**: Photographs of the right (**A**) and left (**B**) fundus show significant regression of exudates and optic disc edema, with the persistance of some peripapillary exudates. **C-D**: Macular OCT scans of the right (**C**) and left (**D**) eyes show complete resolution of serous retinal detachment (SRD), but persistent hyperreflective intraretinal exudates and some disruption of the external limiting membrane

Six months after initiating antihypertensive drugs, visual acuity had progressively recovered, reaching 20/20 in both eyes, along with partial regression of the exudates (Fig. 3). After multidisciplinary discussion, a decision was made to proceed with left nephrectomy.

## Discussion and conclusions

This case illustrates how severe pediatric hypertension can present as neuroretinitis due to the classic combination of optic disc edema and star-shaped macular exudates on initial fundus examination. Because hypertension is rare in children, it may be missed, particularly when ocular findings mimic inflammatory or infectious neuroretinitis.

*Bartonella henselae* is the most frequently implicated pathogen in neuroretinitis, accounting for 65% of cases in some series [3]. Fundus findings include optic disc edema associated with macular exudates in star configurations, as observed in our patient. Macula exudation appears approximately two weeks after the onset of optic disc [4]. However, vitreous cells are sometimes present [5], which was not the case in our patient.

Besides, there were no flame-shaped hemorrhages, only rare cotton-wool patches, and no significant arteriovenous crossing abnormalities, which are classically described in hypertensive retinopathy [6].

Nevertheless, alternative diagnoses must be considered, especially in bilateral presentations, where conditions such as arterial hypertension or intracranial hypertension may be responsible, even though a normal brain MRI is less supportive of the latter hypothesis. Although hypertensive retinopathy is rare in children, it carries a high risk of visual loss and systemic complications if unrecognized [7, 8]. It has been reported in approximately 8% to 18% of hypertensive children, depending on the series [9, 10]. Hypertensive chorioretinopathy is even rarer in pediatric population [11]. It is associated with acute severe hypertension. Importantly, it can be the initial manifestation of an underlying systemic disease, including renal pathology.

Among secondary causes of pediatric hypertension, renovascular hypertension (RVH) accounts for approximately 5 to 25% [12, 13], with delayed diagnosis being associated with poor renal and cardiovascular outcomes. Notably, the most common cause of unilateral renal atrophy associated with hypertension in children is vesicoureteral reflux (VUR) [14], which is responsible for up to 25% of pediatric hypertension cases involving renal damage. According to pediatric guidelines, the therapeutic goal is to maintain blood pressure below the 90th percentile [15]. First-line treatment includes ACE inhibitors or angiotensin receptor blockers, calcium channel blockers, or thiazide diuretics. Nephrectomy is reserved for complex cases of severe secondary renovascular hypertension that are refractory to medical treatment and not amenable to angioplasty. Several cases have described partial or even complete remission of hypertension following nephrectomy [16, 17]. In our patient, early initiation of antihypertensive treatment allowed full visual recovery and significant improvement in cardiac function.

In this case, the peculiarity lay in the fact that blood pressure had initially been recorded as normal during the first hospitalization, despite repeated measurements using several devices, which contributed to a delay in diagnosis. Moreover, a significant diurnal variation in blood pressure has been described in most pediatric populations, with a peak occurring in the late morning that may affect measurements [18, 19]. A subsequent measurement revealed severe hypertension. This highlights that a single normal measurement does not rule out hypertensive disease in children and that repeated assessment is essential in the presence of bilateral disc edema, even when neurological imaging is normal. Furthermore, our case confirms that a hypoplastic kidney functioning poorly and contributing less than 10% of total renal function can act as a trigger for renin-mediated hypertension, and that nephrectomy can be curative when hypertension remains difficult to control medically.

Timely identification and management of hypertensive retinopathy are crucial, as both visual and systemic sequelae may be reversible when treated promptly. This case underscores the need for systematic, repeated blood pressure measurements in all pediatric patients with optic disc edema or neuroretinitis-like presentations, in line with current pediatric hypertension guidelines. It also emphasizes the importance of maintaining a high index of suspicion for renovascular and reflux-associated renal disease, particularly when laboratory findings indicate renin–aldosterone system activation.

## Data Availability

Not applicable.

## Abbreviations

ACE: Angiotensin-converting enzyme
BCVA: Best-corrected visual acuity
OCT: Optical coherence tomography
ICG: Indocyanine green
MRI: Magnetic resonance imaging
FLAIR: Fluid attenuated inversion recovery
eGFR: Estimated glomerular filtration rate
DMSA: Dimercaptosuccinic acid
RVH: Renovascular hypertension
VUR: Vesicoureteral reflux
